# Perceptions of women, their husbands and healthcare providers about anemia in rural Pakistan: Findings from a qualitative exploratory study

**DOI:** 10.1371/journal.pone.0249360

**Published:** 2021-04-27

**Authors:** Sumera Aziz Ali, Anam Feroz, Zahid Abbasi, Savera Aziz Ali, Ahreen Allana, K. Michael Hambidge, Nancy F. Krebs, Jamie E. Westcott, Elizabeth M. McClure, Robert L. Goldenberg, Sarah Saleem

**Affiliations:** 1 Department of Epidemiology, Mailman School of Public Health, Columbia University, New York City, New York, United States of America; 2 Department of Community Health Sciences, Aga Khan University, Karachi, Pakistan; 3 Department of Nursing, University of Alberta, Edmonton, Canada; 4 Department of Pediatrics, Section of Nutrition, University of Colorado Anschutz Medical Campus, Aurora, Colorado, United States of America; 5 Regional Triangulate Institute International, Research Triangle Park, North Carolina, United States of America; 6 Department of Obstetrics and Gynecology, Columbia University, New York City, New York, United States of America; University of Mississippi Medical Center, UNITED STATES

## Abstract

**Background:**

In Pakistan, there is a dearth of literature on the perceptions of anemia among women of reproductive age (WRA). This study was undertaken to explore the perceptions of women, their husbands, and healthcare providers about anemia, its possible causes, and how anemia impacts maternal and child health in Thatta, Pakistan.

**Methods:**

A qualitative study was conducted in Thatta, Pakistan from September to December 2018. Using a pre-tested semi-structured interview (SSI), we collected data to understand their definitions of anemia through ten focus group discussions (FGDs) with women and their partners and ten primary informant interviews (KIIs) with healthcare providers. We identified six major themes: (I) Knowledge and awareness of anemia, (II) Causes and consequences of Anemia, (III) Dietary practices, (IV) Knowledge and practices regarding the use of iron-folic acid supplements, (V) Factors influencing prevention and control of anemia and (VI) Women’s health behavior. We analyzed the data through thematic analysis using NVivo 10 software.

**Results:**

Most community members were not aware of the term anemia but described anemia as a condition characterized by ‘blood deficiency’ in the body. All study participants perceived anemia as an important health problem tending to cause adverse outcomes among WRA and their children. Study participants perceived gutka (chewable tobacco) consumption as an important cause of anemia. Healthcare providers identified short inter-pregnancy intervals, lack of family planning, poor health-seeking behavior, and consumption of unhealthy food as causes of anemia in the district. Consumption of unhealthy food might not be related to related to a poorer knowledge of iron-deficient foods, but economic constraints. This was further endorsed by the healthcare providers who mentioned that most women were too poor to afford iron-rich foods. All men and women were generally well versed with the sources of good nutrition to be consumed by WRA to prevent anemia.

**Conclusion:**

The findings suggest that the government should plan to develop strategies for poverty-stricken and vulnerable rural women and plan health awareness programs to improve dietary practices, compliance with supplements, and health-seeking behavior among women of reproductive age. There is a need to develop effective counseling strategies and context-specific health education sessions to improve the health-seeking behavior of women and men in the Thatta district of Pakistan. Besides, there is need to address social determinants of health such as poverty that pushes women of poorer socioeconomic strata to eat less nutritious foods and have more anaemia. Therefore, a comprehensive and robust strategic plan need to be adopted by government that focuses not only on the awareness programs, but also aim to reduce inequities that lead to pregnant women eat iron-poor foods, which, in turn, forces them to become anemic.

## Introduction

Anemia among women of reproductive age (WRA) is associated with high maternal, fetal, and infant morbidity and mortality [1]. Anemia occurs when the hemoglobin (Hb) concentration drops below a defined cut-off value, thus, decreasing the blood capacity to transport oxygen to the body [2]. Anemia during pregnancy impairs oxygen delivery to the fetus and interferes with normal intra-uterine growth, thereby resulting in adverse fetomaternal outcomes [3, 4]. A variety of factors can lead to anemia among WRA ranging from consumption of an inadequate diet to several infectious diseases (e.g. hookworm infestation, malaria, tuberculosis, HIV) as well as chronic diseases [5].

South-east Asia such as India, Pakistan, Cambodia, Nepal, Maldives, and Bangladesh carries a high burden of anemia among WRA (41.9%), followed closely by the African and Eastern Mediterranean regions [6]. The prevalence of anemia among WRA is consistently higher in people with low socioeconomic status, low body weight, and in females who have recently given birth [7]. According to a National Nutritional Survey of Pakistan (2018), 41.7% of WRA are anemic, with a slightly higher proportion of anemic WRA in rural areas (44.3%) as opposed to 40.2% in the urban areas [8]. More specifically, 18.2% of WRA are iron deficient, which is more common in rural (18.7%) than urban areas (17.4%) [8]. The province of Sindh has the highest proportion of iron deficiency anemia (23.8%), followed by Balochistan (19.0%) and Punjab (18.7%) [8]. Another published study reported the prevalence of anemia to be 75% in one of the regional districts of Pakistan [9]. Likewise, a study conducted in one of the rural areas of Pakistan revealed that 77% of WRA are affected by anemia; of this 20.8% of the WRA are mildly anemic, and more than half (56.5%) of the WRA are affected by moderate to severe anemia [10].

There are huge implications of anemia as it can adversely affect the health of women and children [11]. The consequences of anemia vary according to the type and severity of anemia among WRA [12, 13]. Several studies have shown that anemia among pregnant women can result in poor maternal and fetal outcomes such as abortion, intrauterine growth retardation, post-partum hemorrhage, stillbirths, low-birth-weight, prematurity, and perinatal mortality [3, 14–16]. For instance, a review of observational studies found a linear association between maternal anemia and maternal mortality, with each 10 g/L increase in maternal hemoglobin associated with a 29% reduction in maternal mortality [17]. Likewise, findings from a systematic review revealed that 25% of low birth weight babies, 44% of preterm deliveries, and 21% of perinatal mortality are attributable to anemia during pregnancy in low- and middle-income countries (LMICs) [18]. Furthermore, a meta-analysis showed an increased risk of preterm birth among women who experienced anemia in the first trimester with an overall odds ratio of 1.32 [19]. Most recently, a study based on the WHO multi-country survey demonstrated that severe anemia is associated with a two-fold increase in the risk of maternal death [20]. Similarly, in rural areas of Pakistan anemia is associated with adverse health effects such as postpartum hemorrhage, preterm delivery or stillbirth, and low birth weight babies [10]. Further, a study conducted by Hambidge et al. 2019 found that anemia is a significant and potentially changeable risk factor for birth outcomes among rural Pakistani women in rural Pakistan [21]. Despite the available data on the burden and magnitude of anemia in rural Pakistan, there is a dearth of literature on understanding the communities’ perceptions about anemia, its causes, consequences, and preventive measures. The evidence suggests that if we could decrease the burden of anemia, we could avert around 3190 disability-adjusted life years (DALYs) in the short and long-term [22]. To do that we need to understand the perceptions of those most affected by anemia as well as those stakeholders who provide treatment to prevent or cure anemia. Although there is some evidence on the predictors of anemia among Pakistani women [9, 23, 24], there is a very limited understanding of the perceptions of women, their spouses, and healthcare providers about anemia generally in Pakistan and particularly in the rural areas. The available evidence regarding anemia is mostly from large urban areas of Pakistan instead of rural areas where the prevalence of maternal anemia is higher with limited access to health care facilities [25, 26]. Further, rural and urban areas differ in the social, contextual, and dietary factors, and studies conducted in urban areas do not reflect the knowledge, behavior, and perceptions of rural women [27]. Therefore, studies of urban populations may not be relevant in the rural context of Pakistan. Hence, we undertook this study in the rural area of Pakistan to explore the perception of the community about anemia. Exploring community perceptions is a crucial step towards understanding the contextual factors that influence women’s health-seeking behaviors about anemia. Increased knowledge regarding anemia would help public officials and public health practitioners to design feasible, sustainable, cost-effective, and targeted interventions to reduce anemia among WRA. Therefore, we aimed to conduct this study to explore the perceptions of women, their husbands, and healthcare providers about anemia, its possible causes and consequences, and factors influencing the health behavior of women in Thatta, Pakistan.

## Materials and methods

### Study design and setting

We used an exploratory qualitative research design to understand the perceptions of rural community people about anemia in Pakistan [28]. The study was conducted in one of the rural districts of Sindh, Pakistan. Thatta is situated in the south of Sindh province, adjoining the two largest cities of the province (Karachi, and Hyderabad) [29]. There are 30 union councils (UCs), 3850 villages, 95675 households with an approximate population of 661,517 in the Thatta district [30]. The UC is the smallest administrative unit in a district, with a population of 10,000 people on average and each UC is equipped with a government primary health facility [31]. Since 2008, the Aga Khan University (AKU) in collaboration with the Global Network for Women’s and Children’s Health has maintained a Maternal Newborn Health Registry (MNHR) in 9 UCs of Thatta District, where research is being carried out to improve maternal and child health [32]. For the current study, we sampled separate groups of men and women from 9 UCs of Thatta, where the MNHR [32] is currently available and where subjects were enrolled and gave blood samples for the Women First (WF) trial [33, 34]. We conducted focus group discussions (FGDs) and key informant interviews (KIIs) in different villages of the 9 UCs and at the work stations of healthcare providers (private clinics and centers) respectively.

### Eligibility criteria for study participants

The eligibility was contingent on being married women of reproductive age (15 to 49 years) and their husbands, who could speak the local language (Sindhi) and were residing in the 9 UCs were invited to participate in the FGDs. Likewise, healthcare providers who were either gynecologists, medical doctors, midwives, or traditional birth attendants (TBAs) and had been providing healthcare services in 9 UCs for at least 5 years were included. Participants who did not provide written informed consent were not included in the study. We decided to include women or men if their spouses did not show a willingness to participate. In the protocol, we made it clear that if a woman/man’s participation should be independent of their spouse’s participation. If a woman planned not to participate or vice versa, we approached their spouse and explored their willingness regardless of their partner’s participation. However, we did not face this situation in the field and all women and their husbands showed a willingness to participate and provided written informed consent.

### Sampling technique and sample size

We used a purposive sampling technique to identify and select the study participants to have a diverse sample of women, their spouses, and healthcare providers from the Thatta district. To avoid the selection bias, we recruited women, their spouses, and health care providers from all 9 UCs to have a good representation of participants. To have a wide range of perceptions, we selected participants both from rural and urban geographic areas of the district. We conducted 10 FGDs (5 with males and 5 with females) and 10 KIIs each across 9 UCs of the district Thatta.

### Interview guide

To explore the experiences and perceptions of the study participants, we developed separate semi-structured interview (SSI) guides to conduct FGDs and KIIs with study participants. These interview guides were developed based on the literature search and expert opinion of researchers in the field of qualitative research [35–37]. The interview guide consisted of different sections such as knowledge about anemia, its signs and symptoms, causes of anemia, adverse maternal and child outcomes of anemia, preventive measures, and strategies taken by the government to improve anemia in the district. We pretested the interview guide before the actual research to identify any deficiencies and rectify the same before administering the interview guide on actual study participants. The pretesting was done by the principal investigator and co-investigator (Sumera Aziz Ali (SAA) and Anam Feroz (AF)) in the field and this pretesting took around one week including administration of questions, correction of flow, sequence, and coherence of questions. The interview guide consisted of open-ended semi-structured questions and probes to explore in-depth perceptions of the study participants about anemia.

### Data collection

The principal investigator and co-investigator (Sumera Aziz Ali (SAA) and Anam Feroz (AF)), conceptualized the study and designed the interview guide. However, SAA mainly administered the SSI guides to the women and healthcare providers in the field. The research team comprised of one principal investigator (Assistant professor), one co-investigator (senior instructor), one research coordinator, and two field supervisors. The principal investigator (Female) is a medical doctor and has done a masters in epidemiology and fellowship in community medicine with expertise in maternal and child health. Co-investigator (Female) has a nursing background with masters in health policy management and expertise in qualitative research. The research coordinator (Male) is a social scientist with more than 15 years of experience working in community research with a specific focus on qualitative research. Field supervisors (both females) were from the local community of Thatta and were familiar with the language and culture of the community members. Since we all had been working for at least 10 years in the same community, therefore, are familiar with the culture and norms of the community.

Given the culture of the community and to have the unbiased perceptions of women and their husbands separately, we conducted FGDs of women and their husbands in two different groups at two different places. Thus women could talk in the group and share their opinions without any hesitation. The FGDs of female participants were conducted and moderated by SAA in the household of a woman or TBA where all women study participants were gathered for the interview by providing them the facility of transportation. Similarly, another co-investigator (Zahid Abbasi (ZA)) conducted the FGDs mainly with male participants at a meeting place named "autaq" in the local language where males of the community sit and chat with each other in their free time. We also provided transportation to male participants to join the FGD at a common meeting place. However, SAA and ZA visited healthcare providers in person and conducted KIIs with them at their working stations or clinics, and visited the TBAs at their homes for KIIs. Besides, SAA mainly supervised the data collection and also observed the FGDs of male participants. During the FGDs and KIIS, a free flow of information was encouraged, using probes from these discussions to explore the perceptions in greater detail. Interviews were conducted in person in Sindhi and were audio-recorded after taking the informed consent from study participants. We took field notes in detail during each interview to capture non-verbal language and cues. KIIs lasted continued for 20 and 40 minutes, whereas FGDs lasted 30 minutes to 1 hour and consisted of 8 to 15 participants per group. FGDs and KIIs were audio-recorded, transcribed, and translated into English by a professional translator, checked for accuracy by two authors (SAA) and (AF) both fluent in English and Sindhi.

### Analysis

Data were collected and analyzed through an iterative process. Following transcription of the audio recordings, codes were formulated. Focus groups and KIIs were coded as one data set and an agreement was sought on a coding framework. We coded all transcripts independently according to the agreed outline. Two authors (SAA & AF) discussed and resolved coding discrepancies to minimize researcher bias. We analyzed the codes under major themes. We used the SSI guides to outline and review major themes. We conducted a thematic analysis using NVivo version 10 software.

### Ethical considerations

The Aga Khan University Ethical Review Committee (2018–0262–342) reviewed the protocol of the study and approved the same. The eligible study participants provided written informed consent before collecting data from them. We also took permission from study participants to audio record the interviews and use anonymized quotes. We informed participants about the voluntary nature of their participation and they were given complete right to ask any questions or withdraw from the study at any time during data collection.

## Results

### Characteristics of study participants

Using a pre-tested semi-structured interview (SSI) guide, we collected data through ten focus group discussions (FGDs) with women and their spouses, and ten key-informant interviews (KIIs) with healthcare providers from September to December 2018. Ten FGDs were conducted with several participants in each FGD (Table 1) and ten KIIs were conducted with healthcare providers, one KII per health care provider (Table 2). The number of study participants varied from 7 to 12 for male FGDs, while 10 to 15 women participated in the female FGDs as shown in Table 1. There were 103 participants in 10 FGDs and 10 health care providers in KIIs. All included participants were between 18 and 45 years old, their education status ranged from nil to matriculation (Grade 10) and had a minimum of one child and a maximum of eight children (Table 1). Please see Tables 1 and 2 for an overview of the interview/focus group participants.

**Table 1:** Characteristics of the study participants of focus group discussion (FGDs = 10; the number of participants = 103).

FGD ID	Number of Participants per FGD	Age—Range	Education	Range of children
FGD-M-01	8	19–42	Nil—Intermediate	2–7
FGD-M-02	8	24–45	Nil—Inter Intermediate	2–6
FGD-M-03	8	25–43	Nil—Middle	3–7
FGD-M-04	7	18–42	Nil—Primary	3–6
FGD-M-05	12	20–40	Nil—Middle	1–8
FGD-FM-01	15	18–40	Nil—Matric	1–6
FGD-FM-02	12	18–45	Nil	1–6
FGD-FM-03	11	18–40	Nil	1–6
FGD-FM-04	10	18–40	Nil	2–5
FGD-FM-05	12	18–45	Nil—Primary	2–6

FGD-M: Focus group discussion of males.

FGD-FM: Focus group discussion of females.

**Table 2:** List of participants for key-informant interviews n = 10.

S.No	KII-#	Designation of the Healthcare Provider	Sex of the Health care provider
1	KII-I	Gynecologist	Female
2	KII-2	Medical Doctor	Male
3	KII-3	Medical Doctor	Female
4	KII-4	Staff Nurse	Female
5	KII-5	Midwife	Female
6	KII-6	Traditional Birth Attendant	Female
7	KII-7	Traditional Birth Attendant	Female
8	KII-8	Traditional Birth Attendant	Female
9	KII-9	Traditional Birth Attendant	Female
10	KII-10	Manager Non-Government organization	Male

KII: Key informant interview.

**Fig 1:**
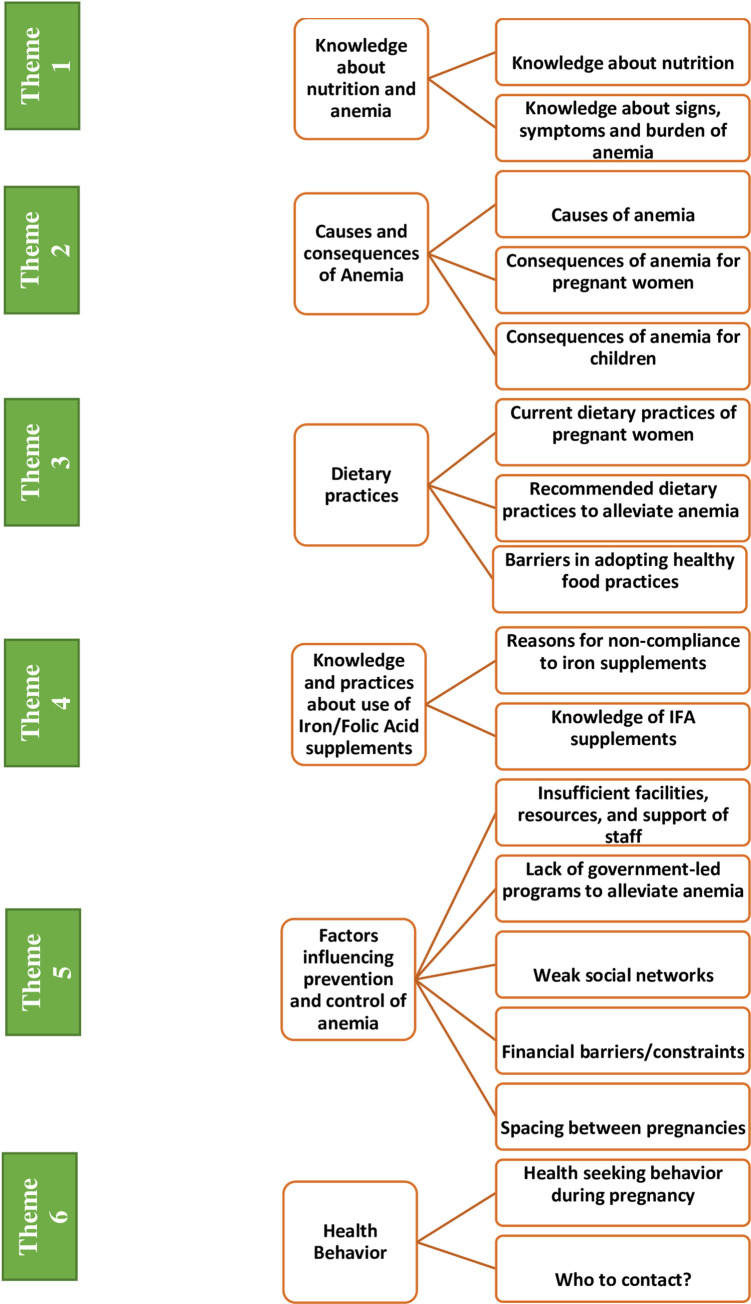
The themes and sub-themes identified in the qualitative study.

The thematic analysis identified six overarching themes: (I) Knowledge about nutrition and anemia, (II) Causes and consequences of anemia, (III) Dietary practices, (IV) Knowledge and practices regarding use of iron-folic acid (IFA) supplements, (V) Factors influencing prevention and control of anemia and (VI) Health behavior as shown in Fig 1. These themes are described below with illustrative quotes.

### Theme 1: Knowledge about nutrition and anemia

#### Knowledge about nutrition

Men described nutrition as ‘good and hygienic food’ and identified fish, meat, chicken, fruits, vegetables, yogurt-based drinks, and butter as sources of good nutrition. They expressed that impurities in food and drinking water can pose a health hazard. The majority claimed not to know much about anemia but felt able to tell from the physical appearance of a woman who appeared weak that it was likely due to ‘blood deficiency’. However, women lacked awareness about health maintenance and the need to seek medical assistance when necessary.

They stated that the diets of pregnant women should include fruits such as apples, bananas, and pomegranates, and vegetables including spinach, cauliflower, okra, eggplant, and ridge gourd. Processed fruit juices were not encouraged. Unlike urban women, women from rural areas had a good intake of fish since their husbands’ primary occupation was fishing.

“*We belong to the Sindhi culture so we mostly eat fish instead of vegetables and meat” (FGD-F-01)”*.

Most women were not aware of anemia and described it as ‘deficiency of blood in the body’ or ‘weakness’. Some described it as a condition that requires medical treatment including blood transfusion as per the advice of a healthcare provider.

“*Blood is synthesized from food and water and when we eat 10 grams of food then body forms 1 drop of blood”(FGD-F-02)*.

According to healthcare providers, some women considered anemia/blood deficiency to be a physiological process and not a disease.

"*Women think that blood deficiency is a normal process*, *which occurs during delivery and the older women (in the community) tell them that it is not a disease or a health problem"(KII-HCP-02)*.

#### Knowledge about signs, symptoms, and burden of anemia

Healthcare providers reported that most community women had obvious signs and symptoms of anemia such as fatigue, pallor, leg cramps, and edema. They carried out physical examinations to look for signs of anemia followed by a blood hemoglobin test to determine its severity and start necessary treatment. They believed most hemoglobin levels to be lower than 8 gm/dl due to frequent pregnancies.

“*For every 100 women we assess*, *almost 60 to 80 are anemic and if I measure it on a scale of 10*, *then 8 out of 10 women are anemic” (KII-HCP-02)*.

They highlighted that 99% of pregnant women in the area were anemic and that anemia was more common in women than men. In addition, the vast majority of these women presented to clinics with Hb levels of 3 to 4 gm/dl during their pregnancy and low hemoglobin was the most common reason for specialist referrals. Some women reported not having enough energy for their daily activities as well as feeling short of breath, experiencing shivering, pallor, and gastric burning due to anemia. Some talked about how they could identify anemia by looking at the nail beds and eyes, which develop pitting and pallor respectively. According to them, anemia could have an adverse effect on their vision and also cause tachycardia.

"*We generally get the idea of blood deficiency from the hands and eyes of women as they become pale*. *Women feel weak and are unable to perform daily activities*. *Upon checkup*, *doctors ask for some blood tests and inform us that they are anemic" (FGD-M-01)*.

“*Due to blood deficiency, our blood pressure also goes down, and we cannot perform any work” (FGD-F-02)*.

### Theme 2: Causes and consequences of anemia

#### Causes of anemia

Doctors in KIIs and males in FGDs reported that intake of chewable tobacco [Gutka/Mawa/Chura in the local language] was a major cause of anemia among pregnant women. They noted that women in the district were addicted to chewable tobacco (gutka), which damages the mucosal membrane and prevents iron absorption. Gutka also reduced their appetite, preventing them from consuming a nutritious diet, consequently making them weak and anemic.

Some women also reported leading far more stressful lives than their male counterparts who were sometimes away from home for days on end, leaving them solely responsible for the care of their children. However, we did not find health care providers and males who reported stress as a cause of anemia among WRA.

“*Our husbands come home like guests but we have to stay at home with our children all the time*. *These stressors make us weaker” (FGD-F-05)*.

Besides, men reported that early marriages were very common in rural areas resulting in multiple pregnancies at a young age predisposing young females to anemia. Furthermore, the doctors stated that most males residing in rural areas did not consider anemia to be a serious health issue.

Additionally, consumption of fuller’s earth [multani mud in the local language] was also considered to be an indirect cause of anemia as it decreased the consumption of a nutritious diet. Women in the FGDs identified strenuous fieldwork performed by pregnant women as a potential cause of anemia. However, strenuous work was not identified as a cause of anemia by men or health care providers. Other factors that TBAs and healthcare providers enumerated were illiteracy, lack of knowledge about the causes of anemia, and limited antenatal follow-ups. Working barefoot in the fields was also identified as a causative factor as it could provide a favorable route for worms to penetrate the skin of the feet and enter the body.

Women with short inter-pregnancy intervals who did not consume a balanced diet were more likely to be anemic. Similarly, those who were engaged in strenuous field-work were left with little time to focus on their diet. Healthcare providers noted that breastfeeding mothers who did not consume a nutritious diet were at a significantly greater risk of developing anemia.

"*We are addicted; we will not eat meat but we will buy gutka even though gutka is more expensive than medicines" (FGD-F-05)*.

"*In the villages*, *women have a lot of workloads; they work barefoot in the fields all day*, *I think that is the reason why they get blood deficiency*. *The heat that enters the body from their feet has an adverse effect on their eyes and causes their blood to dry as well " (KII-HCP (TBA)-06)*

“*Women who stay at home are healthier and their blood levels normal” (FGD-F-03)*.

"*In the present time*, *a major cause of anemia is frequent pregnancies; each female has almost 9 to 10 children" (KII-HCP-01)*.

"*God knows how many children a woman produces because usually*, *they do not tell us how many children are alive*. *I have received a patient who has 23 gravidae with six to seven miscarriages*. *Is this not the reason for becoming anemic*?*" (KII-HCP-02)*.

"*Here in rural areas*, *early marriages are very common and this is our biggest mistake*. *We just fix their marriages once they reach puberty*. *We don’t think if they are mature enough to take up the responsibility or not*. *In urban areas*, *people send their girls to school so that they gain awareness but here we don’t do that " (FGD-M-02)*.

#### Consequences of anemia for pregnant women

Women expressed that anemia caused them to experience vertigo and weakness. Additionally, some women believed that anemia may cause pregnancy complications including seizures, headache, dehydration, life-threatening bleeding, necessitating a Cesarean delivery. Healthcare providers highlighted the strong association between a hemoglobin level of less than 5gm/dL and postpartum hemorrhage.

“*If she is anemic and has blood deficiency then she will not be able to deliver the child through a normal delivery and will have to undergo a major surgery*, *which can cause death” (FGD-F- 02)*.

#### Consequences of anemia for children

Concerning child development, the consensus among women was that anemia resulted in stunted growth and inability to gain adequate weight. Besides causing miscarriage and stillbirth, anemia during pregnancy could also be a potential cause of premature birth, low birth weight, congenital anomalies, and malnutrition in the offspring. Males in the FGDs and healthcare providers voiced their concerns about growth retardation and emphasized that women should have a nutritious diet to avoid long-term complications in the offspring.

“*With the inadequate transfer of nutrients from the mother to the developing baby*, *the child will become ill" (FGD-M-03)*.

"*The child may die in the mother’s womb due to blood deficiency in the woman” (FGD-F-05)*.

### Theme 3: Dietary practices

#### Current dietary practices of pregnant women

According to most couples, while pregnant women in urban settings were encouraged to consume protein-rich foods including fish, meat, and pulses, those in the rural areas had a diet similar to that of non-pregnant women because of financial constraints. On inquiring about the number of meals per day, women from rural areas mentioned that they only consumed food when hungry.

They highlighted that tobacco makes up 80% of the pregnant woman’s diet and was considered a regular grocery item. The consumption of fuller’s earth by pregnant women was another major cause of anemia within this subset.

Healthcare providers noted that most pregnant women believed that tobacco consumption was associated with energy and strength. The participants further explained that unfortunately these behaviors and habits got passed on to their children as well. When a young boy asked his parents for gutka, they would take pride in the fact that their sons were old enough to do so.

They also reported that despite having easy access to vegetables grown on their farms, most women preferred selling them in the market to make money rather than consuming it themselves.

"*Eating gutka is very common in women*. *Every mother eats gutka herself as well as feeds it to the baby that is still in her lap" (KII-HCP-01)*.

"*In villages*, *children who do not even have a complete set of teeth have been eating gutka" (KII-HCP-02)*.

#### Recommended dietary practices to alleviate anemia

Women in the FGDs mentioned that vegetables such as spinach, eggplant, bottle gourd, and okra and fruits such as apples, bananas, and pomegranates are essential to alleviate anemia.

Healthcare providers recommended that pregnant mothers eat with other family members rather than eating leftovers at the end as well as increase the intake of available resources such as cucumbers and dates. They proposed consuming a bowl of spinach a day to meet the daily iron requirements, stating that it has the same nutrition value as injectable iron.

“*1 ampule of Jectofer (iron injection) is equal to 1 bowl of Spinach and if you take that instead*, *it will save the doctor’s fee*, *transportation expense*, *and also be more beneficial because it’s natural food”(KII-HCP-03)*.

"*In winters*, *family members give dates and oil to their cattle but don’t give it to the women" (KII-HCP-02)*.

#### Barriers in adopting healthy food practices

On inquiring about barriers encountered in adopting healthy dietary habits, men stated that the poor quality food available in the markets could be a potential reason for women developing anemia during pregnancy. The use of tap water instead of purified drinkable water was another cause. One TBA highlighted that medication was not always used to treat anemia among pregnant women because of limited financial resources.

They also noted that over the years, people in the community preferred selling milk and eggs instead of consuming it themselves. They used the money to purchase unhealthy foods such as gutka for their family members.

"*People coming from cities collect all the fresh items from villagers and give them a schedule for the next delivery’” (KII-HCP (TBA)-02)*.

### Theme 4: Knowledge and practices regarding the use of Iron–Folic Acid (IFA) supplements

#### Knowledge of IFA supplements

Men and women in the FGDs mentioned that doctors and TBAs advised them to take ‘small yellow tablets’ (folic acid), ‘black tablets’ (ferrous sulfate), ‘syrups’ (syngobion), and ‘red or brown capsules’ for anemia. The healthcare providers mentioned that they provided iron and folic acid supplements from the government hospital medication supply to the women, but not all women used the supplements.

“*We have invested too much time and money to visit your clinic not to get these tiny and useless tablets” (KII-HCP-02)*.

#### Reasons for non-compliance to iron supplements

TBAs reported that limited financial resources, lack of awareness, and illiteracy are the major reasons for non-compliance to iron and folic acid supplements during pregnancy. The health care providers also mentioned that women residing in the city areas also do not consume their medications regularly. They purchase the medications or supplements from the store or government health care facility but do not consume the supplements regularly. Furthermore, the TBAs expressed that pregnant women do not visit healthcare facilities unless there is a real need for blood transfusion. They warned the husbands to take better care of their wives who were anemic because if not controlled, it might have serious health complications, necessitate transfusion, or even cause death. Unfortunately, however, the response that they usually received was that they still did not perceive anemia as something serious and would visit the clinic for a transfusion if need be. The doctors mentioned that the lady health workers (LHWs) provided iron and folic acid supplements to the women at their doorstep but did not follow up to ensure compliance. In addition, doctors in public hospitals did not have enough time to counsel women.

*“Even educated people of urban areas are not smart; they buy iron during pregnancies but don’t take those medications” (KII-HCP-01)*.

"*Even if a couple has lost 8 children in the past*, *it is not an issue for them as they believe they can produce another one*. *God forbid if*, *during the ninth pregnancy the woman loses her life*, *her husband would not worry about it because he would consider it God’s will" (KII-HCP (TBA)-01)*.

### Theme 5: Factors influencing prevention and control of anemia

#### Insufficient facilities, resources, and support of staff

The study participants reported that medicines were often unavailable at hospitals and nearby stores. Similarly, the hospitals charged heavily for most laboratory tests which they could not afford. When blood products were required for transfusion during delivery, it was usually very difficult to arrange them because of a dearth of blood banks in the district. The paramedical staff also demanded money as their commission (under-the-table payment), which put an additional burden on the family.

The healthcare providers noticed how most women who visited the clinics for their antenatal visits only underwent an ultrasound and were sent back without any prenatal counseling. If these doctors were to perform a thorough screening for anemia and provide adequate treatment as well as nutritional counseling, they could potentially prevent women from becoming anemic.

Healthcare providers expressed concerns about the risk of infection in women who came to the clinic for blood transfusions because of a lack of sterilization techniques employed by these centers. They were also at risk of acquiring blood-borne infections from the transfused blood products such as hepatitis B and HIV because of limited screening facilities. Transfusion without screening in a non-emergency setting is imprudent and could also be life-threatening.

They stated that many healthcare providers were not trained to interpret investigation findings such as a complete blood count of the patients. It is impossible for them to then diagnose and treat anemia.

"*When we visit hospitals for C-sections*, *the woman may sometimes die due to bleeding because we cannot afford blood transfusion*. *She can only get blood if her family can afford to pay the cost of the blood products otherwise most people living here cannot afford it and so the women lose their lives" (FGD-M-02)*.

"*Nowadays*, *doctors assess around 300 to 400 patients per day in their clinics*, *which is fine*, *but at least they should take out the time to guide and counsel them properly" (KII-HCP-01)*.

*Most of the blood banks in the district are not registered and provide unscreened blood to patients; we are not even sure if we are provided with human blood” (KII-HCP-01)*.

#### Lack of government-led programs to alleviate anemia

The lack of government support for the health sector means that public hospitals oftentimes fail to provide adequate facilities and quality care to families who can not afford treatment in private hospitals. Emergency room admissions also incur a massive fee, that most people from rural settings can not afford. The study participants highlighted that the government’s efforts to improve healthcare facilities have mostly targeted urban hospitals while the rural population continues to stay neglected. Most of the study participants also highlighted that the government needs to take immediate action to ban the use of gutka and other non-nutritious food items that are less expensive and accessible to the men and women in the communities. Healthcare providers expressed the failure of the government to invest in anemia- specific programs and recommended that they undertake counseling sessions to sensitize the population about adequate dietary intake and family planning. Indeed, the government has arranged training sessions to address other obstetrical problems in the past but unfortunately, anemia during pregnancy is not one of them. Therefore, it should take the responsibility to actively train doctors as well as educate the population with the help of awareness sessions and other campaigns.

“*Government should ban the use of gutka and other harmful items that cause blood deficiency so that people can save money to buy healthy things" (FGD-F-01)*.

#### Weak social networks

Women complained about a lack of social support from their community leaders who were power and money-oriented and would only reach out to them when they needed their support to win elections. Once that was done, there was nobody to pay heed to their health concerns and if they required medical assistance, they would have to arrange transport and manage the logistics themselves. These comments of women about the lack of prioritization to facilitating healthcare resonate with their comments on field-work and the absence of men in theme 2.

“*You are the only one asking us about our health and interviewing us regarding this; no one else is working on health problems” (FGD-F-05)*.

"*Community leaders do not even bother even if a woman dies in the village*. *They simply just forget about the deceased woman and move on with life" (FGD-F-02)*.

#### Financial barriers/constraints

Both men and women highlighted the need for better employment opportunities to be provided by the government. This would ensure financial security and enable them to improve the quality of their lives. Equally important was the need for healthcare providers to provide medicines free of cost.

One of the women from the cohort expressed that she had been anemic for the past six months and upon consulting the doctor, was advised a drip costing 2000 rupees ($12). Unfortunately, this was out of her reach. The healthcare providers noted that most patients approaching them were too poor to afford fruits or iron-rich food.

“*Wealthy families can afford blood transfusion for the treatment of blood deficiency among women but poor people cannot afford it” (KII-HCP-06)*.

"*When doctors refer us to the hospitals*, *we have to take a loan from others to get treatment" (FGD-F-05)*.

#### Spacing between pregnancies

Most study participants did not believe in family planning (FP) or birth spacing and thought it was normal to get pregnant every year. They also avoided contraceptives (especially the injectable forms) which were perceived to be associated with complications such as bleeding, weight gain, and dizziness. However, a handful of participants did admit that birth spacing was beneficial for the health of both the mother and the child.

Doctors reported that people in the district had several myths regarding FP. It also seemed to go against their religious and cultural beliefs. Thus religion was identified as the main barrier to use FP methods; however, women themselves did not directly state religion as a barrier to use FP. For instance, health care providers mentioned that post-partum FP was considered ‘sinful’ by most mothers. Healthcare providers thought that modern methods of FP would be fruitless unless awareness about their usage was created amongst the masses and their myths and misconceptions were addressed. Indirectly, health care providers considered lack of awareness as a cause behind disbeliefs towards pregnancy spacing. Cultural beliefs make it essential to counsel women in the absence of their husbands who typically express disapproval over FP methods. However, we did not find religion or resistance from men as reasons regarding disbeliefs towards pregnancy spacing.

They recommended the use of FP methods especially if the couple did not have the financial means to provide basic needs such as food, shelter, and education to their children, and also suggested permanent contraception such as tubal ligation and vasectomy when the family was complete.

“*The woman who is getting pregnant is perceived to be better than the woman who is doing family planning” (FGD-F-05)*.

"*As soon as her first child starts walking*, *the woman will try for another baby" (FGD-F-05)*.

"*God will be unhappy if you discuss family planning with me and His unhappiness will result in the death of my child" (KII-HCP-02)*

### Theme 6: Health behavior

#### Health seeking behavior during pregnancy

The poor health-seeking behavior of the women in the community meant that they sought medical treatment only when severely anemic and were often not compliant with treatment. Pregnant women visited the clinics during the 2^nd^ trimester with Hb levels of 6 to 7 gm/dL when they would be prescribed parenteral iron until delivery.

Additionally, most women were also not compliant with antenatal visits, investigations, and follow up visits after delivery—only 40% returned for post-partum follow-ups. The women, on the other hand, blamed this on the doctors who did not give them quality time during their visits. This controversy between the community and health care providers could be either true or it might be natural human behavior to blame others for their mistakes. However, we could not explore the reasons for this controversy and it might be possible that health care providers might have their limitations such as work overload or flow of patients given the higher population in the district. On the other hand, community people might not visit the health care providers on time due to lack of resources as highlighted by men, women, and TBAs.

“*During a*n*tennal care*, *women only want to get their ultrasound done to determine the sex of the baby rather than being concerned about the health of their child" (HCP*, *KII- 01)*.

Furthermore, the men stated that if someone got sick, it took them up to three days to visit the doctor and start treatment. The doctors in private clinics also charged a higher fee than the ones in government facilities and so they avoided visiting them.

"*When we go to a government hospital*, *doctors tell us that they also practice in "Doctors Street" in the evening and so I should come to visit them in their private clinic where they would perform some tests and start treatment” (FGD-M-Chatochand)*.

#### Who to contact?

Women in the FGDs mentioned seeking pregnancy care from the local TBAs and doctors who also provided medicines. Complicated pregnancies were referred to the public and private facilities for pregnancy care and childbirth. They often self-treated themselves using home-based remedies or visited homeopathic doctors or spiritual healers.

"*We go according to our affordability*, *sometimes we don’t go to the hospital we just deliver the baby at home" (FGD-F-05)*.

## Discussion

This study identified the perceptions of women, their husbands, and healthcare providers about anemia, its risk factors, and perceived causes, its impact on maternal and child health, and factors affecting the prevention and control of anemia in district Thatta, Pakistan. Most community members were not aware of the term anemia but described anemia as a condition characterized by ‘blood deficiency’ in the body, which sometimes necessitated a blood transfusion. All study participants perceived anemia as an important health problem tending to cause adverse outcomes among WRA and their children. In addition, study participants perceived gutka consumption as one of the important causes of anemia in this population. Healthcare providers identified short inter-pregnancy intervals, lack of FP, poor health-seeking behavior, and consumption of unhealthy food as causes of anemia in the district. All men and women were generally well versed with the sources of good nutrition to be consumed by WRA to prevent anemia. These findings should help raise relevant issues to be addressed by health-care planners in developing countries, such as Pakistan, which has a very high prevalence of anemia. Findings of the study provide a unique opportunity to better understand community members’ and healthcare providers’ perceptions about anemia, and design context-specific strategies and interventions to prevent and control anemia during the reproductive cycle of women residing in the rural areas of Pakistan [28].

Our study findings are consistent with other studies conducted in South Asia. For instance, a cross-sectional, descriptive study conducted at Sri Manakula Vinayagar Medical College Hospital, India revealed that despite recognizing the symptoms of anemia, women were not familiar with the term ‘anemia’ (60.13%) [38]. Healthcare providers indicated that women perceived anemia as ’normal during pregnancy’ and considered weakness and dizziness as the usual symptoms of pregnancy. This is also similar to the findings of a study on anemia conducted in Mumbai, India [36], where women considered anemia to be a typical symptom of pregnancy as they believed that the pregnant woman’s body had to share nutrients and blood with the fetus [36]. This finding reflects a lack of awareness of women about anemia and its symptoms mainly during pregnancy. This further indicates the need to design some education strategies to raise awareness among women about this misconception about anemia being a normal phenomenon during pregnancy. The government of Pakistan has a well-established Lady health program, where LHWs visit pregnant women at their doorsteps and provide health education and iron supplements to women during pregnancy [39]. Although LHWs provide health education, there should be a separate component of awareness regarding anemia, its symptoms, risks for both mothers and their babies, and its implications for a family if not treated on time.

Almost all of the study participants identified gutka as one of the major causes of anemia among women of reproductive age in the district of Thatta, Pakistan. This is a new finding, which has not been reported earlier in comparable studies. Gutka is a type of smokeless chewable tobacco made up of areca nut, slaked lime, catechu, tobacco, flavoring agents, and sweeteners [40]. The literature highlights that biochemically, tobacco use may affect iron metabolism, iron stores, inflammation, and hemoglobin levels [41–43]. Behaviorally, the use of tobacco may suppress appetite and has been associated with lower food intake and household food insecurity [44, 45]. Hence, tobacco found in gutka suppresses the appetite, reducing the intake of food and important micronutrients by WRA, thereby increasing their susceptibility to anemia [46]. Gutka has made inroads in traditional society and people of lower socioeconomic status as an alternative to smoking [47]. The myth that gutkha is less harmful than smoking poses a major challenge [48]. Gutka consumption has increased because of aggressive advertisements, and packed sachets, which are available in various brands in almost all shops at an affordable cost [49].

In addition to gutka consumption, TBAs and healthcare providers also perceived consumption of fuller’s earth, illiteracy, lack of awareness about the causes of anemia, limited antenatal checkups, frequent pregnancies with short inter-pregnancy intervals, working barefoot in the farms as primary causes of blood deficiency. Similar studies in Ethiopia and India support our findings [50–54]. For instance, literature has revealed that an inter-pregnancy interval of fewer than 2 years weakens the process of anatomical and physiological recovery after delivery, thus increasing the chances of anemia in subsequent pregnancies [55, 56]. Similarly, based on the above findings, hookworm infection seems to be closely related to women working barefoot in the field. In the body, hookworms parasitize the proximal small intestine, leading to chronic intestinal hemorrhage and iron deficiency anemia [57]. They can also cause severe gastrointestinal bleeding, especially in the setting of inadequate iron stores of the host, thus resulting in anemia [57].

Our findings further demonstrate that despite counseling about the consumption of a nutritious diet, women in the Thatta district have poor health-seeking behaviors and continue to consume gutka. Similar findings were reported by a study conducted at an Australian tertiary obstetric hospital, where women were counseled on a variety of issues relating to the prevention and treatment of anemia; but despite that, there was a lack of compliance [58]. Therefore, further research is needed to understand both incentives and barriers to improving the health behavior of women and perceptions with respect to compliance with treatment [58].

Healthcare providers considered a lack of awareness, illiteracy, unavailability of medicines at government facilities, and financial constraints as some of the reasons for women consuming unhealthy diets and their poor compliance with IFA supplements. To ensure adequate coverage of antenatal IFA supplementation, a study conducted in Pakistan recommended interventions such as providing adequate awareness, good quality counseling, reminder messages, and free supplements throughout pregnancy [59]. The healthcare providers also noted that most women visiting their clinics were too impoverished to afford fruits or iron-rich foods. This is a striking finding and alluded to broader social and contextual factors that prevail in the society rather than at an individual level. Poverty or low purchasing power is a broader social rather than individual-level factor that needs to be addressed as a fundamental cause of the disease [60]. For example, we can educate women about the dietary habits they must adopt as individuals by consuming a healthy diet to overcome anemia. Such a strategy will be futile because regardless of how much awareness women have about the risks of anemia, some women may be deprived of financial, physical, or social resources that would enable them to change their eating habits. This implies that the burden of disease can not be eliminated unless interventions address broader social factors that shape the behavior of individuals. Such social factors can be either addressed by improving the education status of girls to make them self-sufficient or by providing job opportunities to women or their spouses to enable them to purchase iron-rich foods. This would help to have sustainable and long-term impacts on the overall health of women.

Another important issue highlighted by healthcare providers was the risk of infections such as hepatitis B and HIV among women who required blood transfusions. Acquiring infections via blood transfusion is prevalent in developing countries and is mostly attributed to a lack of screening and sterilization facilities. Such risk is higher for women who are severely anemic and have limited options to improve their anemia status on an urgent basis [61]. This is an alarming finding and calls for urgent action. Given the higher burden of anemia, the need for blood transfusion can not be avoided mainly for pregnant women, and access to safe blood transfusion is considered a global priority. This is further reflected by the Model list of Essential medicines by the World Health Organization (WHO), where blood components are considered as essential medicines [62]. This implies that authorities need to emphasize the strict monitoring and supervision of the quality, safety, and efficacy of the blood transfusion [63]. The local government of Pakistan must take the necessary steps to ensure availability as well as the accessibility of blood transfusion products. Such blood products should be screened adequately, and are stored and transfused under sterilized conditions to avoid infections, thereby addressing the burden of anemia without increasing unnecessary risk of infection among women.

Our study also points out some of the major barriers hindering the prevention and control of anemia. These include insufficient facilities and resources, lack of support from staff, lack of government-led programs to alleviate anemia, weak social networks, financial barriers/constraints, and lack of birth spacing. For example, one of the alarming findings of the study was the myths and misconception about FP, where mothers considered FP as sinful. These findings are analogous to the studies conducted in other Muslim developing countries where religion is one of the major barriers to FP usage [64, 65]. However, in most of the Muslim countries, Islam supports contraception usage [66]. The literature reveals that religious leaders can play a vital role to improve the uptake of contraception by advocating for FP. For example, A study conducted in Nigeria observed that messages delivered by religious leaders regarding modern FP methods enhanced uptake by Muslim women [67]. Likewise, religious leaders have been considered as change agents to bring change in attitudes towards FP among Somali Muslim women [68]. Given the powerful ranks of religious leaders in Muslim countries like Pakistan, the strategies to enhance uptake of FP methods should involve religious leaders to advocate for FP.

### Strengths and limitations

This study is the first of its kind that has explored the perceptions of women, their spouses, and healthcare providers about the most important and relevant public health problem in rural Pakistan. We tried to have diverse opinions of the healthcare providers mainly the TBA who are very close to the women in rural areas and who also understand the health problems of rural women. Lastly, the principal investigator was involved actively in all stages of the study ranging from the conceptualization of the study to analysis of the data. This supervision and monitoring ensured the quality and richness of the data collected in the field. However, our study has some limitations. First, we only included married women of reproductive age and did not include single mothers or pregnant adolescents. Second, the analysis of the interviews was based on the subjective accounts of the respondents, and therefore cannot be generalized to other settings. Lastly, due to limited funds, we could not interview healthcare providers such as LHWs and staff of Basic Health Units who are an important part of the government healthcare delivery system in Pakistan. Therefore, future studies should consider having the perceptions of anemia of these important stakeholders in rural areas of Pakistan.

## Conclusion

Anemia is a major public health concern in rural Pakistan. The study revealed that most men and women were not aware of the term "anemia", but they did know that women of reproductive age suffer from blood deficiency. Study participants identified gutka as one of the major causes of anemia among women of reproductive age in the district of Thatta, Pakistan. Frequent pregnancies with small inter-pregnancy intervals were identified as an important cause of anemia by healthcare providers. Additionally, the health-seeking behavior among WRA was found to be unsatisfactory, as indicated by poor dietary practices, lack of compliance with iron-and folic acid supplements, and excessive consumption of gutka.

This study has several policy implications for the rural communities of Pakistan. The findings of the study suggest that the government should plan to develop safety nets for poverty-stricken women and initiate health awareness programs to improve dietary practices, compliance with supplements, and health-seeking behavior among women of reproductive age. Furthermore, there is a need to develop effective counseling strategies and conduct context-specific health education sessions to improve the health-seeking behavior of women and men in Thatta district, Pakistan. More specifically, the government should target disadvantageous and vulnerable women with limited access to healthcare facilities and Pakistan’s LHW program can play a vital role to provide knowledge and health education about preventive measures for anemia to women at their doorsteps. Unfortunately, we could not interview LHWs in our study and we acknowledge that LHWs are considered as a bridge between the broader health system and women. Therefore, before making any concrete recommendations about improving access to health care, we strongly recommend taking perceptions of LHWs about anemia and their role in preventing the same. Without understanding perceptions of LHWs in future studies, it might be premature to comment on access to care. However, this should not stop us to make recommendations for government to arrange frequent health education sessions and campaigns for the community people to create awareness about the anemia both for the local people of the communities as well as the health care providers. Also, given the high illiteracy rate of women in Thatta Pakistan, there is an urgent need for health and education sectors to work in collaboration to improve the overall health of rural Pakistani women. Education of girls will not only increase awareness among them regarding behavior change but will also empower them to make healthy choices independently and thereby affect change for the better. Thus, future educational strategies should be developed to promote gender equality in all areas of education in Pakistan, but particularly to increase female students’ participation in education. Besides, there is need to address social determinants of health such as poverty that pushes women of poorer socioeconomic strata to eat less nutritious foods and have more anaemia. Therefore, a comprehensive and robust strategic plan need to be adopted by government that focuses not only on the awareness programs, but also aim to reduce inequities that lead to pregnant women eat iron-poor foods, which, in turn, forces them to become anemic.

## Supporting information

S1 File Interview guide—qualitative anemia study. (DOCX)
