# Supplementary material for: Perceptions of women, their husbands and healthcare providers about anemia in rural Pakistan: Findings from a qualitative exploratory study
Source: PLoS One. 2021 Apr 27;16(4):e0249360. doi: 10.1371/journal.pone.0249360 (PMC8078764; doi:10.1371/journal.pone.0249360)
Supplement: S1 File — (DOCX) [file pone.0249360.s001.docx]

**Risk factors of anemia among women of reproductive age group in Thatta district: Women First Anemia study**

**Note: This guide will be administered by qualified and technical people, e.g PI of the study.**

**Focus group discussion guide for participants**

**Demographics**

Age عمر

Family Size/Number of Children گهر ۾ ڀاتين/ٻارن جي تعداد

Husband’s Job مڙس جو پيشو

Literacy/Years of Schoolingتعليم

1. What do you know about nutrition? توهان غذائيت جي باري ۾ ڇاٿاڄاڻو؟

Probes

- In what ways/from whom did you learn about nutrition?
- Have you received nutritional information?

1. Does the health of a mother affect her child’s health? ڇا ماءِ جي صحت ٻار جي صحت تي اثرانداز ٿئي ٿي

Probes

- How?

1. What does a woman’s diet consist of? عورتن جي غذا ۾ ڪهڙيون شيون شامل آَهن؟

Probes

- Do you feel that you have a balanced diet? Are you eating any special or traditional foods during your pregnancy?
- How frequently do you eat green leafy vegetables?

1. What do you understand by anemia?رت جي ڪمي يا اينميا جي باري ۾ توهان ڇا ٿا ڄاڻو؟

Probes

- A problem of blood in women
- Low level hemoglobin in pregnancy
- Low oxygen carrying capacity of blood
- Low volume of blood

1. In your opinion, what causes anemia among pregnant women?

توهان جي خيال ۾ عام طرح حامله عورتن ۾ رت جي ڪمي ڇو ٿيندي آهي؟

Probes

- Mosquitoes
- Bleeding
- Not eating well
- Worm infestation
- Genetic disease

1. In your perception, what are the locally predominant factors causing anemia among non-pregnant women?توهان جي خيال ۾ عام طرح حامله عورتن ۾ رت جي ڪمي جا ڪهڙا سبب آهن؟
2. What are the sign and symptoms of anemia? رت جي ڪمي جو اندازو ڪئين لڳائي سگهجي ٿو؟

Probes

- Dizziness
- Easy fatigability
- Fever
- Weakness
- Palpitations

1. In your opinion, what are the effects of anemia in pregnancy on the unborn child?

توهان جي خيال ۾ رت جي ڪمي وارن عورتن جو ٻارن تي ڪهڙو اثر پوي ٿو؟

Probes

- low birth weight
- intra-uterine death
- congenital anomalies

1. In your opinion, what are the effects of anemia in pregnancy on the mother?

توهان جي خيال ۾ حامله عورتن ۾ رت جي عمل سان ڪهڙو اثر ٿي سگهي ٿو؟

Probes

- Body weakness
- Maternal death
- Stroke

1. Are you aware, whom does one need to contact for anemia?

ڇا توهان ٻڌائي سگهو ٿا ته رت جي ڪمي جي لاءِ ڪنهن سان رابطو ڪجي؟

Probes

- Doctors
- LHVs

1. Do you know what iron is? Are you taking supplements currently?

آئرن جي باري۾ ٿوهان ڇا ٿا ڄاڻو؟ توهان اهڙي قسم جون دوائون استعمال ڪريه پيا؟

- Where did you get them?
- Did you take them when you were pregnant?
- Why are you taking the supplements/what are they for? Do you take them with water or milk?

1. In your opinion, why a woman should take these supplements?

توهان جي خيال ۾ عورتن کي اهڙي دواون جو استعمال ڇو ڪرڻ گهر جي؟

1. In your opinion, what women should include in their diet?

توهان جي خيال ۾ عورتن جي غذا ۾ ڪهڙيون شامل ڪيون وڃن؟

1. In your opinion, what local community perceive regarding the prevention of anemia?

رت جي ڪمي کان پڇڻ لاءِڪميونٽي جا ڪهڙا خيال آهن ؟

1. In your opinion, what Government should do to address the problems of anemia?

توهان جي خيال ۾ گورنمنٽ کي رت جي ڪمي جي حوالي سان ڪهڙو ڪردار ادا ڪرڻ گهر جي؟

1. In your opinion, do you see any relationship of the socio demographic, economic, and cultural dimensions with anemia?

توهان جي خيال ۾ سماجي، معاشي ۽ ثفاقتي جو ڪو ڪردار آهي رت جي ڪمي جي حوالي سان؟

1. In your opinion what should be the minimum interval between births?

توهان جي خيال ۾ ٻارن ۾ گهٽ ۾ گهٽ ڪيترو وقفو هئڻ گهر جي؟

**In-depth Interview guide for health care provide (Nurse/Midwife/TBA)**

- - - 1. What are the major health problems faced by women in your communities?

توهان جي ڪميونٽي ۾ عورتن جا صحت جا مسئلا ڪهڙا آهن؟

- - - 1. What do you about know anemia (mainly from TBA)?

رت جي ڪمي جي باري ۾ توهان ڇا ٿا ڄائو؟

- - - 1. In your opinion, what proportion of women do you think present with anemia in your clinics or facilities?

توهان جي خيال ۾ توهان جي ڪلينڪ ۾ رت جي ڪمي سان ايندڙ عورتن جو تناسب ڪيترو آهي؟

- - - 1. What do you think about the causes of anemia in your communities?

توهان جي خيال ۾ رت جي ڪمي جا سبب ڪهڙا آهن؟

- - - 1. In your opinion, what type of women is at risk of anemia?

توهان جي خيال ۾ ڪهڙي عورتن ۾ رت جي ڪمي جو انديشو هوندو آهي؟

- - - 1. What do you think about the difference of Hemoglobin levels between men and women of your community?

عورتن ۽ مردن ۾ رت جي ڪمي جي حوالي سان ڪافي فرق آهي ، توهان جو ان متعلق ڇا خيال آهي؟

- - - 1. In your opinion, what measures do you take to address this problem?

توهان جي خيال ۾ انهن مسئلي کي ڪيئن حل ڪري سگهجي ٿو؟

- - - 1. What are your perceptions regarding your competencies to manage pregnant women with anemia?

توهان رت جي ڪمي وارن عورتن جو علاج ڪرڻ لاءِ جيڪا هجڻ گهرجي ان جي باري ۾ ڇا خيال آهي؟

- - - 1. In your opinion, what are perception and experience of patients’ and their families’ regarding anemia?

توهان جي خيال ۾ رت جي ڪمي وارن عورتن جي خاندان وارن جا ڪهڙا تاثرات هوندا آهن؟

- - - 1. Do you think, community people identify anemia as a major problem or to what extent they give importance to this problem?

توهان جي خيال ۾ ڪميونٽي وارن جي لاءِ رت جي ڪمي وڏو مسئلو آهي؟

- - - 1. In your opinion, what type of actions should community take to tackle the problem of anemia?

توهانجي خيال ۾ ڪميونٽي کي انهن مسئلي جي لاءِ ڇا ڪرڻ گهرجي؟

- - - 1. What do you think about the availability of supporting resources such as medical facilities and human resources at the Health Centers to address this crucial problem of anemia?

توهان جي خيال ۾ رت جي ڪمي جي حل لاءِ ڪهڙيون سهولتون موجود آهن؟

- - - 1. Do you think there is any relationship between Family planning usage and anemia?

توهانجي خيال ۾ خانداني منصوبه بندي جو رت جي ڪمي سان ڪو تعلق آهي؟

- - - 1. In your opinion, what type of interventions should be designed by Government for anemia?

توهان جي خيال ۾ گورنمنٽ کي رت جي ڪمي جي لاءِ ڇا ڪرڻ گهر جي؟

- - - 1. Any other message or comment you want to convey?

توهان انهن متعلق ٻيو ڪجهه چوڻ چاهيندا؟
